# Diagnostic accuracy of MRI in local staging of rectal cancer, and determining the surgical resection margin status; retrospective study. The experience in Oman

**DOI:** 10.1186/1470-7330-14-S1-P9

**Published:** 2014-10-09

**Authors:** AMA Al-Hadidi, A Kamona, M Al Qubtan, J Al Kalbi, S Al-Tai, MS Al-Hanashi

**Affiliations:** 1 Oman Medical Specialty Board, Royal Hospital, Oman

## Aims and objectives

To retrospectively review the accuracy of magnetic resonance (MR) imaging in preoperative staging of rectal cancer and to predict surgical circumferential resection margin status, with histopathologic results

## Methods and materials

MR images of 39 patients (15 underwent surgery without pre-operative treatment, while 24 had undergone pre-operative CRT or radiotherapy prior to MRI) with diagnosis of rectal cancer to evaluate tumour stage (T stage), involvement of mesorectal fascia (MRF), and nodal metastasis (N stage) were included in the study. Tumours were staged according to the TNM staging system (American Joint Committee on Cancer guidelines)

## Results

We managed to correctly determine T stage in 86.6%, and 70.8%, correctly assess the status of MRF in

100%, and 70.8%, and correctly determine the N stage in 66.6%, and 54.1%, for the first and second group, respectively.

## Conclusion

Main T staging errors contributed to desmoplastic reaction in the first group, or post-therapy changes in the second group, which also affected the MRF.

The limited accuracy of nodal size is likely to be related to the fact that 30%–50% of metastases in rectal cancer occur in nodes that are less than 5 mm.

MRI is the primary method for local staging of rectal cancer mainly due to high soft tissue resolution which makes it better in evaluating tumour extension, relation to adjacent pelvic organs, and also for the ability to assess the MRF. Results are highly dependent on the technique of the examination, which needs meticulous care and standardization.

